# PPARγ alleviates peritoneal fibrosis progression along with promoting GLUT1 expression and suppressing peritoneal mesothelial cell proliferation

**DOI:** 10.1007/s11010-022-04419-y

**Published:** 2022-04-05

**Authors:** Junxia Feng, Meizhi Lu, Wenhao Li, Jingchun Li, Ping Meng, Zukai Li, Xuejuan Gao, Yunfang Zhang

**Affiliations:** 1 Department of Nephrology, Affiliated Huadu Hospital, Southern Medical University (People’s Hospital of Huadu District), 48 Xinhua Road, 510800 Guangzhou, China; 2 The Third School of Clinical Medicine, Southern Medical University, Guangzhou, China; 3 Key Laboratory of Functional Protein Research of Guangdong Higher Education Institutes and MOE Key Laboratory of Tumor Molecular Biology, Institute of Life and Health Engineering, Jinan University, Guangzhou, China

**Keywords:** PPARγ, Peritoneal fibrosis, Peritoneal dialysis, GLUT, Glucose transport

## Abstract

**Objective:**

Peritoneal fibrosis (PF) is commonly induced by bioincompatible dialysate exposure during peritoneal dialysis, but the underlying mechanisms remain elusive. This study aimed to investigate the roles of peroxisome proliferator-activated receptor gamma (PPARγ) in PF pathogenesis.

**Methods:**

Rat and cellular PF models were established by high glucose dialysate and lipopolysaccharide treatments. Serum creatinine, urea nitrogen, and glucose contents were detected by ELISA. Histological evaluation was done through H&E and Masson staining. GLUT1, PPARγ, and other protein expression were measured by qRT-PCR, western blotting, and IHC. PPARγ and GLUT1 subcellular distribution were detected using confocal microscopy. Cell proliferation was assessed by MTT and Edu staining.

**Results:**

Serum creatinine, urea nitrogen and glucose, and PPARγ and GLUT1 expression in rat PF model were reduced by PPARγ agonists Rosiglitazone or 15d-PGJ2 and elevated by antagonist GW9662. Rosiglitazone or 15d-PGJ2 repressed and GW9662 aggravated peritoneal fibrosis in rat PF model. PPARγ and GLUT1 were mainly localized in nucleus and cytosols of peritoneal mesothelial cells, respectively, which were reduced in cellular PF model, enhanced by Rosiglitazone or 15d-PGJ2, and repressed by GW9662. TGF-β and a-SMA expression was elevated in cellular PF model, which was inhibited by Rosiglitazone or 15d-PGJ2 and promoted by GW9662. PPARγ silencing reduced GLUT1, elevated a-SMA and TGF-b expression, and promoted peritoneal mesothelial cell proliferation, which were oppositely changed by PPARγ overexpression.

**Conclusion:**

PPARγ inhibited high glucose-induced peritoneal fibrosis progression through elevating GLUT1 expression and repressing peritoneal mesothelial cell proliferation.

## Introduction

Peritoneal dialysis (PD) has been widely applied for treating end-stage renal disease (ESRD) patients as a common replacement therapy and other renal disorders such as chronic kidney diseases [1–3]. Globally, more than 272,000 ESRD patients were reported to undergo peritoneal dialysis treatment each year which accounted for nearly 11% of total dialysis patient population [2, 4]. The successful application of PD greatly depends on the morphologic and functional integrity of peritoneal membrane (PM) composed of the mesothelial monolayer and the submesothelial compact zone, which serves as the semipermeable barrier during PD treatment [3]. Unfortunately, long-term exposure to bioincompatible PD solutions such as high glucose and AGEs (advanced glycation end products) usually causes peritoneal fibrosis (PF), featured by the loss of mesothelial cells (MCs) and thickening of submesothelial layer and regarded as one major reason for peritoneal ultrafiltration failure [3, 5]. Inhibition of mesothelial cell proliferation was also suggested as potential strategy of attenuating PF progression [6]. However, the molecular mechanism underlying high glucose-induced mesothelial cell functional alterations and PF development remains poorly understood.

GLUT1 (glucose transporter type 1) is one major transmembrane glucose transporter protein encoded by the SLC2A1 (Solute carrier family 2 member 1) gene, whose protein pore domain contains twelve transmembrane segments and intracellular terminus [7]. Also, aberrant expressional and functional changes of GLUT1 were prevalently associated with the development of various fibrosis-related disorders including renal fibrosis in diabetic nephropathy and hepatic stellate cell activation during liver fibrosis [8, 9]. Moreover, GLUT1 was previously shown to be implicated in the alleviation of peritoneal dialysis-induced peritoneal fibrosis and encapsulating peritoneal sclerosis progression by glucocorticoid treatment [10]. The expression of GLUT1 in peritoneal mesothelial cells could be significantly altered by high glucose level, associated with glucose toxicity and chronic ambulatory peritoneal dialysis (CAPD) [11]. The pathogenic roles of GLUT1 in PD-associated with PF development, as well as its up-stream regulatory mechanisms, deserve further investigations.

PPARγ (peroxisome proliferator-activated receptor gamma) is one of the ligand-activated transcription factor that belongs to the nuclear receptor protein superfamily [12]. The cellular PPARγ could be activated by various ligands such as 5-oxo-eicosatetraenoic acid and other arachidonic acid metabolites, as well as multiple artificial drugs like Rosiglitazone, GW1929, 15d-PGJ2, and GW2090 [13, 14]. In consistence with its pleiotropic biological functions, PPARγ has also been characterized as one critical player in distinct human disorders, especially fibrosis pathogenesis in cardiac, renal, pulmonary, and other human tissues [12]. The activation of PPARγ with specific agonists has also been regarded as one promising way of inhibiting fibrosis development. For instance, PPARγ agonist pioglitazone was recently shown to suppress TGF-β (tumor growth factor beta)-induced glomerulosclerosis and renal fibrosis [15]. Moreover, the activation and elevated expression of PPARγ contributed to the suppression of high glucose/PD-induced PF by pioglitazone and Telmisartan in rat model and peritoneal mesothelial cells [16, 17]. More importantly, PPARγ could also regulate the expression of GLUT1 to modulate renal tubule homeostasis and renal tubulointerstitial fibrosis [18]. However, the relationship between PPARγ and GLUT1 underlying PD-induced PF pathogenesis is still elusive.

In this study, we aimed to investigate the activity of PPARγ and its effects on the GLUT1 expression in regulating high glucose-induced peritoneal fibrosis pathogenesis using both rat and peritoneal mesothelial cell models, which might be further explored for preventing and treating patients with PD-induced PF.

## Material and methods

### Animals model and treatments

Six-week-old male SD rats with a body weight of 200 ± 15 g purchased from the Experimental Animal Center of the Southern Medical University were raised in autoclaved animal cages at 24 ± 2 °C with a ambient humidity of 55% ± 5% and a 12:12 h light–dark cycle. All rats were fed with standard laboratory chow combined with drinking water ad libitum. Totally, 50 male SD rats were randomly divided into the blank (*n* = 10), model (*n* = 10), rosiglitazone (*n* = 10), 15d-PGJ2 (*n* = 10), and GW9662 (*n* = 10) groups. To induce peritoneal fibrosis, rats in the model, rosiglitazone, 15d-PGJ2, and GW9662 groups were administrated with lipopolysaccharide (LPS) (l mg/kg) by intraperitoneal injection, which were then intraperitoneally injected with peritoneal dialysis (PD) solutions (100 mL/kg) containing 25% glucose. For the blank group, the same volume of normal saline solution was daily given to each rat by intraperitoneal injection. At the end of the experiment and under continuous anesthesia, the animals were euthanized by overdose of pentobarbital (125 mg/kg). These experimental operations on rats were approved in advance by the Experimental Animal Care and Use Committee of the Southern Medical University (No. 2021019). The rosiglitazone group was treated with 20 mg/kg rosiglitazone each day by intragastric administration for 4 days before model establishment. Rats of the 15d-PGJ2 and GW9662 groups were intraperitoneally injected with 0.3 mg/kg 15d-PGJ2 and 1 mg/kg GW9662, respectively, each day, lasting for successive 4 days.

### Peritoneal mesothelial cell isolation and culture

Rat peritoneal membrane tissues surgically collected were immediately rinsed three times with pre-chilled PBS solution and incubated with 20 ml cell dissociation buffer containing 0.25% trypsin and 0.015% EDTA for 25 min at 37 °C with supply of 5% CO_2_ and frequent shaking. After passing the stainless-steel wire mesh (100 pore), the cell suspension was mixed with 20 ml RPMI-1640 medium (Thermo Fishier Scientific) containing 20% calf serum. The above cell mixture was then centrifuged at 1000 rpm for 10 min, and the precipitate was re-suspended in RPMI-1640 medium and cultured at 37 °C in a humidified chamber with 5% CO_2_. Twenty-four hours later, the cell culture medium was replaced with the same volume of fresh RPMI-1640 medium, followed by refreshment of culture medium every three days. Normal cell passages were then performed, while the cell confluency in culture dishes reached over 80%.

### Gene silencing

The silencing of PPARγ expression in peritoneal mesothelial cells was finished by small interfering RNA (siRNA) transfection. siRNA targeting PPARγ (siPPARγ: (5′ GCGGAGATCTCCAGTGAUATT-3′) and its control (siNC: (5′ TTCTCCGAACGTGTCACGTTT-3′) were synthesized by the GenePharma Biotech (Shanghai, China), which were introduced into cultured rat peritoneal mesothelial cells using the Lipofectamine 3000 reagent (Thermo Fishier Scientific) following the producer’s instructions. Cells were harvested for following assays 48 h after the transfection.

### Recombinant lentivirus and gene overexpression

The lentivirus-mediated transfection was performed to overexpress PPARγ gene in rat peritoneal mesothelial cells. First, the PPARγ gene coding sequence was amplified by PCR method using the primers PPARγ-CDS-F (5′ GTGGGGATGTCTCACAATGC -3′) and PPARγ-CDS-R (5′ TTTCCTGTCAAGATCGCCCT-3′), which was then inserted into the H125 pLenti-TRE-EGFP-EF1-rtTA3-IRES-Puro (Obio Technology; Shanghai, China). Subsequently, cultured 293T cells were co-transfected with the recombinant H125-PPARγ vector or original H125 vector together with pLP1, pLP2, and pLP-VSVG vectors, using the Lipofectamine 3000 reagent introduced above. Cell culture medium was then collected 72 h later and centrifuged at 4000×*g* for 12 min, followed by filtering through a 0.45 μm membrane to collect lentivirus. Rat peritoneal mesothelial cells cultured in 6-well plates with a confluence of about 80% were added with 15 μL polybrene (1 mg/mL) and the above-mentioned H125 or recombinant virus (2 mL/well), followed by the replacement of 1 mL culture medium with 1 mL McCoy’s 5A culture medium 24 h after transfection and screening with puromycin (12 μL, 1 mg/mL) 72 h later. The expression of PPARγ gene was finally verified by western blotting.

### ELISA (enzyme linked immunosorbent assay)

The creatinine (CRE), blood urea nitrogen (BUN), and blood glucose contents in rat serum after specified treatments were determined by the ELISA (enzyme linked immunosorbent assay) method. Rat serum CRE and BUN level were measured using the commercially available kit (CRE: #C011-2-1; BUN: #C013-2-1) produced by the Nanjing Jiancheng Bioengineering Institute (Nanjing, Jiangsu, China) following the manufacturer’s instructions. The rat blood glucose levels were detected using the Rat GLU ELISA kit produced by the Shanghai win–win Biotechnology Company (Shanghai, China) as instructed by the manufacturer. Three biological replicates were performed for statistical evaluation.

### Quantitative RT-PCR

Total RNA samples from rat peritoneal membrane tissues or cultured rat peritoneal mesothelial cells were prepared using the RNA Easy Fast total RNA extraction kit (#DP451; TIANGEN Biotech, Beijing, China) according to the producer’s instructions. RNA concentrations were measured on a NanoDrop 2000 instrument (Thermo Fishier Scientific). Subsequently, the cDNA samples were synthesized by reverse transcription method using the TIANScript II RT Kit (#KR107; TIANGEN Biotech, Beijing, China) from approximately 5 μg total RNAs of each group. Finally, the expression levels of target genes were determined through the real-time quantitative PCR method using the FastFire qPCR PreMix (SYBR Green) (#FP207; TIANGEN Biotech, Beijing, China) following the protocol by the manufacturer. Expression levels of GAPDH gene were simultaneously detected as the internal standard for the final quantitation of gene relative expression by the standard 2^−∆∆Ct^ method based on at least three biological replicates. The sequences and product lengths of primers used for mRNA quantitation are shown in Table 1.

**Table 1:** The sequences of primers used for quantitating mRNA expression

Gene ID	Primer sequence (5′–3′)	Product length (bp)
GAPDH F	CCTCGTCTCATAGACAAGATGGT	169
GAPDH R	GGGTAGAGTCATACTGGAACATG	
TGF-β F	CTGACCCCCACTGATACGC	255
TGF-β R	CAGGTGTTGAGCCCTTTCC	
GLUT1 F	ATGAAAGAAGAGGGTCGGCA	231
GLUT1 R	GAAGGCCGTGTTGACGATAC	
PPARγ F	GTGGGGATGTCTCACAATGC	203
PPARγ R	TTTCCTGTCAAGATCGCCCT	
a-SMA F	GGGCCAAAAGGACAGCTATG	86
a-SMA R	TGATGCCGTGTTCTATCGGA	

### Western blotting

The total RNA samples of rat peritoneal membrane tissues or rat peritoneal mesothelial cells were extracted using the Western/IP Cell Lysis Buffer (#P0013; Beyotime Biotechnology, Shanghai, China) following the producer’s instructions. Protein concentrations were determined through the BCA method. About 30 μg proteins of each group was boiled at 98 °C for 5 min, separated using 12% SDS-PAGE, and transferred onto PVDF membranes. Following blocking with 5% BSA solution for 2 h at room temperature, the PVDF membrane containing proteins was then incubated with primary antibodies diluted in TBST for overnight at 4 °C, washed three times with TBST, and incubated with HRP-conjugated secondary antibodies for 1.5 h at room temperature. The abundances of proteins were finally determined using the BeyoECL Star ECL kit (#P0018AM; Beyotime Biotechnology, Shanghai, China) according to the manufacturer’s instructions. Relative expression of target proteins was finally assessed by calibration to the GAPDH protein after three biological replicates. Primary antibodies used for western blotting assay in this study contain anti-PPARγ (#ab272718; Abcam), anti-GLUT1 (#12939; CST), anti-a-SMA (#19245; CST), anti-TGF-β (#ab9758; Abcam), and anti-GAPDH (#ab181602; Abcam).

### Hematoxylin and eosin (H&E) staining and immunohistochemistry (IHC)

The rat peritoneal membrane tissues were sliced into 5-μm-thick sections, which were then subjected to fixation with 4% neutral phosphate-buffered formalin and embedded in paraffin. Subsequently, rat peritoneal membrane tissue sections were dewaxed with xylene for 20 min, rehydrated by incubation with 100% ethanol for 20 min, 95% ethanol for 5 min, 70% ethanol for 5 min, and 50% ethanol for 5 min. The antigen retrieval was then done by incubating with 0.01 M citric acid buffer (pH 6.0) at 95 °C for 12 min, followed by incubation with 3% H_2_O_2_ at room temperature for 8 min. After being incubated with 5% BSA solution for 25 min at 37 °C, rat tissue slides were then incubated with anti-PPARγ (#2430; CST; 1: 500) or anti-GLUT1 (#12939; CST; 1:500) for 1.5–2 h at 37 °C or overnight at 4 °C, followed by incubation with HRP-conjugated anti-rabbit IgG antibodies (#ab190492; Abcam; 1:500) for 30 min at 37 °C, and developed with DAB substrate kit (#ab64238; Abcam). Following staining using the Hematoxylin and Eosin Staining Kit (#C0105M; Beyotime Biotechnology), the slides were then dehydrated, mounted, and observed under microscopy.

### Masson staining

The Masson staining method was performed to analyze the collagen fiber levels in rat peritoneal tissues using the Masson's Trichrome Stain Kit (#G1340; SolarBio Life Sciences, Beijing, China) following the producer’s instructions. Briefly, after dewaxed to water, rat tissue slides were stained with hematoxylin solution for 7 min, stained with Masson blue solution for 4 min, washed with distilled water for 1 min, stained with ponceau S/acid fuchsin solution for 6 min at room temperature, washed with phosphomolybdic acid solution for 90 s, stained with aniline blue solution for another 90 s, washed with weak acid solution for 50 s, dehydrated with 95% ethanol and 100% ethanol, cleared with xylene, and finally mounted with neutral balsam. The collagen fiber deposition in rat peritoneal tissues was observed under microscopy based on three biological repeats.

### Immunofluorescence (IF) and laser confocal microscopy

The expressions of Vimentin and Keratin in rat peritoneal mesothelial cells were validated by immunofluorescence method. Briefly, rat peritoneal mesothelial cell slides were prepared, fixed with 4% paraformaldehyde for 30 min at room temperature, treated with 0.2% Triton X-100 for 60 min at room temperature for permeabilization, and blocked with 4% BSA solution for 40 min at room temperature. Subsequently, rat peritoneal mesothelial cell slides were washed with PBS and incubated with the anti-Vimentin (#ab92547; Abcam; 1:500) or anti-Keratin (#ab8068; Abcam; 1:500) for 1.5 h at room temperature and 95% humidity, followed by incubation with fluorescent-conjugated secondary antibodies for 2 h at room temperature. To evaluate the subcellular localization of PPARγ and GLUT1 proteins, rat peritoneal mesothelial cells were treated as above-mentioned using anti-PPARγ gamma (#ab272718; Abcam; 1:500) and anti-GLUT1 (#12939; CST; 1:500) antibodies, which were finally observed using laser confocal microscope.

### Cell proliferation

The proliferation rates of rat peritoneal mesothelial cells were analyzed in this study by both the MTT and Edu staining methods. The MTT assay was carried out using the MTT (#M8180; Solarbio Life Sciences, Beijing, China) as instructed by the manufacturer. Briefly, rat peritoneal mesothelial cells were cultured in 96-well plates, mixed, and incubated with MTS reagent for 0.5–2 h at 37 °C in a humidified chamber with supply of 5% CO_2_ and frequent shaking, followed by measuring the OD490 (absorbance at 490 nm) with a multi-functional plate reader. The evaluation of rat peritoneal mesothelial cell proliferation by Edu staining was finished using the BeyoClick EdU-647 Cell Proliferation kit (#C0081L; Beyotime Biotechnology, Beijing, China) following the manufacturer’s protocol.

### Statistical analysis

At least three biological replicates were performed to produce quantitative data presented as mean ± standard deviation, which were analyzed by the SPSS 20.0 software. Differences between 2 or more groups were evaluated by the student T test or ANOVA (analysis of variance), respectively, using P < 0.05 as the threshold for significant differences.

## Results

### PPARγ inhibits peritoneal fibrosis progression in rat model

**Fig. 1:**
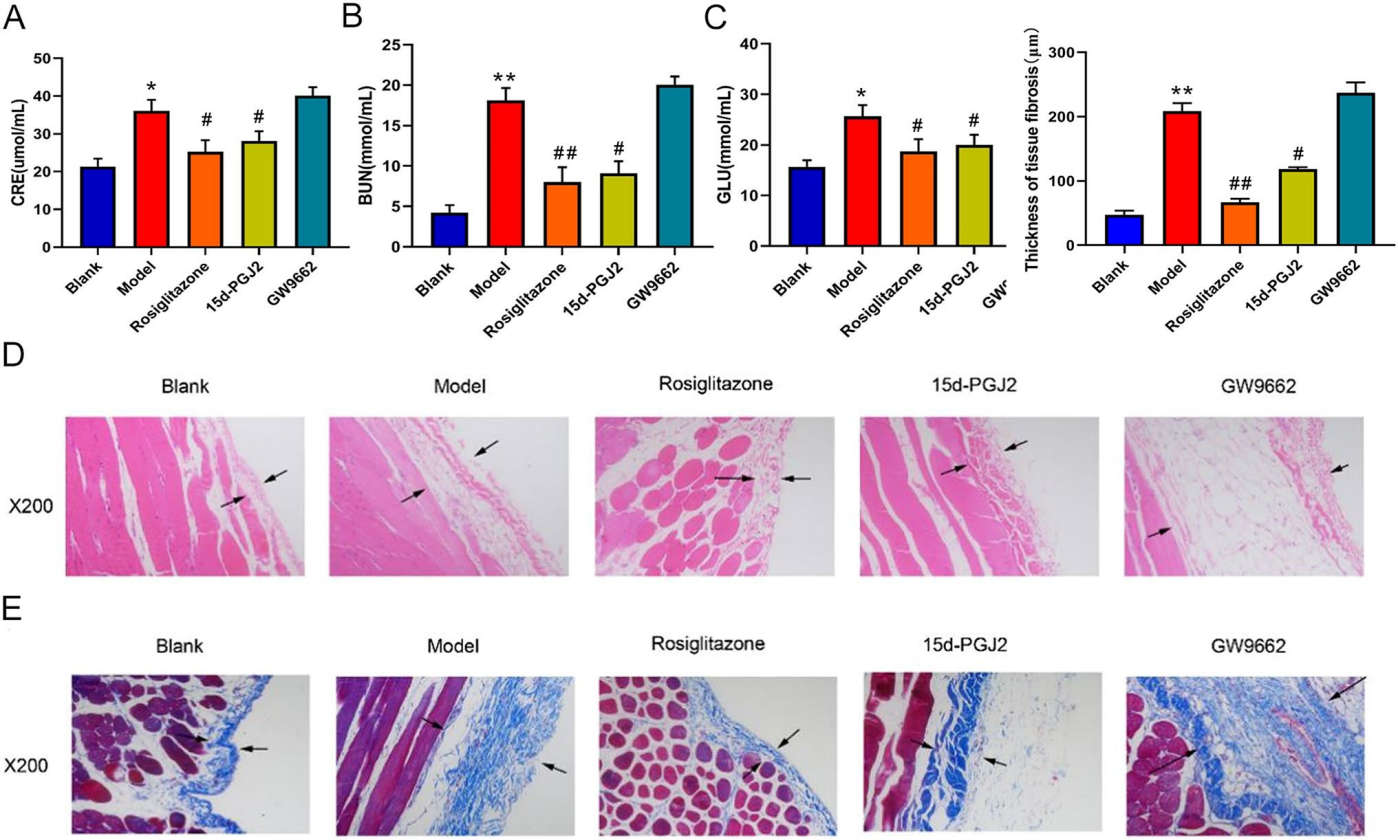
Regulation of rat peritoneal fibrosis progression by PPARγ agonists and antagonist. **A**–**C** The alteration of creatinine (**A**), urea nitrogen (**B**), and blood glucose (**C**) contents in the rat PF model treated with rosiglitazone, 15d-PGJ2, or GW9662. The rat creatinine, urea nitrogen, and blood glucose levels of each group as indicated were determined through the ELISA method. **D** Histological evaluation of the influences of rosiglitazone, 15d-PGJ2, or GW9662 on submesothelial layer thickness changes in the rat PF model of different groups. Rat peritoneal tissues were subjected to hematoxylin–eosin (H&E) staining and observed under microscopy. Black arrows indicate the submesothelial layers. **E** The changes of collagen fiber contents in the peritoneal membranes of rat PF model treated with rosiglitazone, 15d-PGJ2, or GW9662. Collagen fiber levels in rat peritoneal membranes were measured by Masson staining. *CRE* creatinine, *BUN* blood urea nitrogen, *GLU* glucose; *P < 0.05 and **P < 0.01 (vs the blank group); ^#^*P* < 0.05 and ^##^*P* < 0.01 (vs the model group)

To explore the role of PPARγ in modulating peritoneal fibrosis (PF) development, we first established the rat PF model with the combination of high glucose and lipopolysaccharide (LPS) treatments as introduced in methods. We found that the creatinine, blood urea nitrogen, and blood glucose contents in the model rats were significantly increased compared with the blank group (Fig. 1A–C). However, the PPARγ agonists rosiglitazone or 15d-PGJ2 effectively decreased CRE, BUN, and blood glucose levels in the PF model rats, while PPARγ antagonist GW9662 caused no significant changes of CRE, BUN, and blood glucose contents in the model rats (Fig. 1A–C). Through hematoxylin–eosin (H&E) staining, we observed that the thickness of the rat submesothelial layer in the model group was greatly increased compared with the blank group, which was then remarkably decreased by rosiglitazone or 15d-PGJ2 treatment but increased by GW9662 treatment (Fig. 1D). Moreover, Masson staining demonstrated that the collagen fiber content in the peritoneal membrane tissues of the rat model group was also greatly elevated in comparison to the blank group, which was significantly reduced by rosiglitazone or 15d-PGJ2 treatment but severely increased by GW9662 treatment (Fig. 1E). These results showed that PPARγ antagonist rosiglitazone and 15d-PGJ2 alleviated and PPARγ antagonist GW9662 aggravated PF progression in the rat model.

### PPARγ elevates GLUT1 expression in peritoneal membranes of rat PF model

**Fig. 2:**
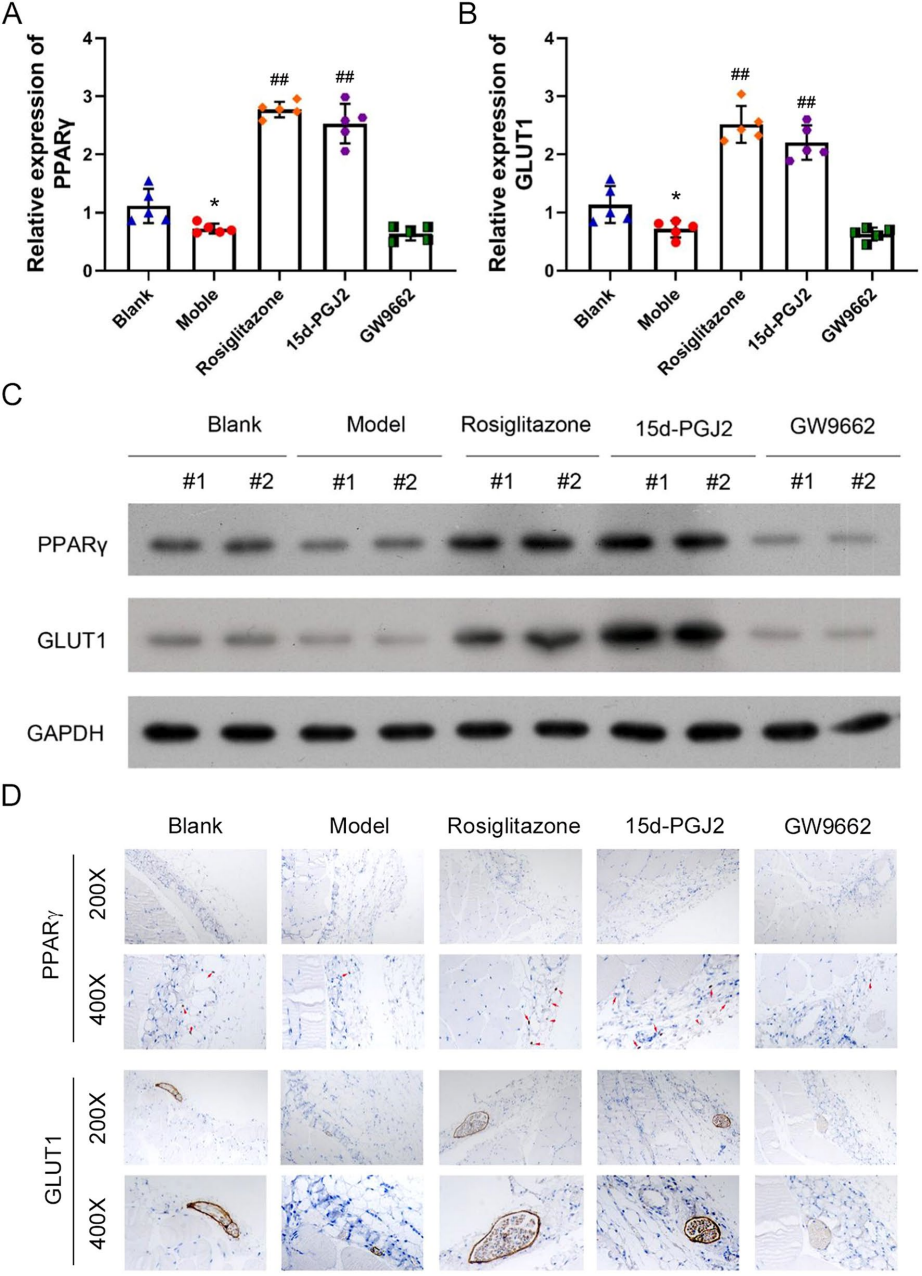
Positive correlation of PPARγ and GLUT1 expression in peritoneal membranes of rat PF model. **A**, **B** Relative mRNA levels of PPARγ and GLUT1 in the peritoneal membrane tissues of rat PF model treated with rosiglitazone, 15d-PGJ2, or GW9662. The quantitative RT-PCR method was performed to quantitate mRNA levels in rat peritoneal membranes. **C** Alteration of PPARγ and GLUT1 protein abundances in peritoneal membrane tissues of rat PF model treated with rosiglitazone, 15d-PGJ2, or GW9662. PPARγ and GLUT1 protein levels in rat peritoneal membrane were measured by western blotting with GAPDH as the internal standard. **D** In situ PPARγ and GLUT1 protein levels in the peritoneal membranes of rat PF model affected by treatment with rosiglitazone, 15d-PGJ2, or GW9662. Immunohistochemistry method was used to detect the in situ expression of PPARγ and GLUT1 in rat peritoneal membrane. *GLUT1* glucose transporter type 1, *PPARγ* peroxisome proliferator-activated receptor gamma, *GAPDH* glyceraldehyde-3-phosphate dehydrogenase; *P < 0.05 (vs the blank group); ^##^*P* < 0.01 (vs the model group)

For more understanding of the molecular mechanisms, we then assessed the interaction between PPARγ and GLUT1 expression in peritoneal fibrosis progression. The expression of PPARγ mRNA was down-regulated in the peritoneal membrane of rat PF model compared with the blank group, and rosiglitazone and 15d-PGJ2 elevated while GW9662 treatment inhibited PPARγ expression in PF rat peritoneal membrane (Fig. 2A). Importantly, we found that the GLUT1 mRNA levels in peritoneal membrane of rat PF model were also lowered compared with the blank group, promoted by rosiglitazone and 15d-PGJ2 treatment, and suppressed by GW9662 treatment (Fig. 2B). Consistent alterations of PPARγ and GLUT1 protein contents were also observed in the peritoneal membrane tissues of PF rat models via western blotting assay (Fig. 2C). The increases of PPARγ protein levels in PF rat peritoneal membranes with rosiglitazone and 15d-PGJ2 treatments resulted in a great increase of GLUT1 protein level as well (Fig. 2C). On the contrary, the GLUT1 protein levels in rat PF model treated with GW9662 treatment were remarkably lower than the model group (Fig. 2C). Furthermore, the promotion of GLUT1 protein level by rosiglitazone and 15d-PGJ2 treatment as well as the down-regulation of GLUT1 protein expression by GW9662 treatment in peritoneal membranes of rat PF model were all verified by the immunohistochemistry (IHC) assay (Fig. 2D). These results showed that GLUT1 protein expression was positively regulated by PPARγ during its regulation of peritoneal fibrosis progression.

### PPARγ promotes GLUT1 expression and inhibits fibrosis in rat peritoneal mesothelial cells.

**Fig. 3:**
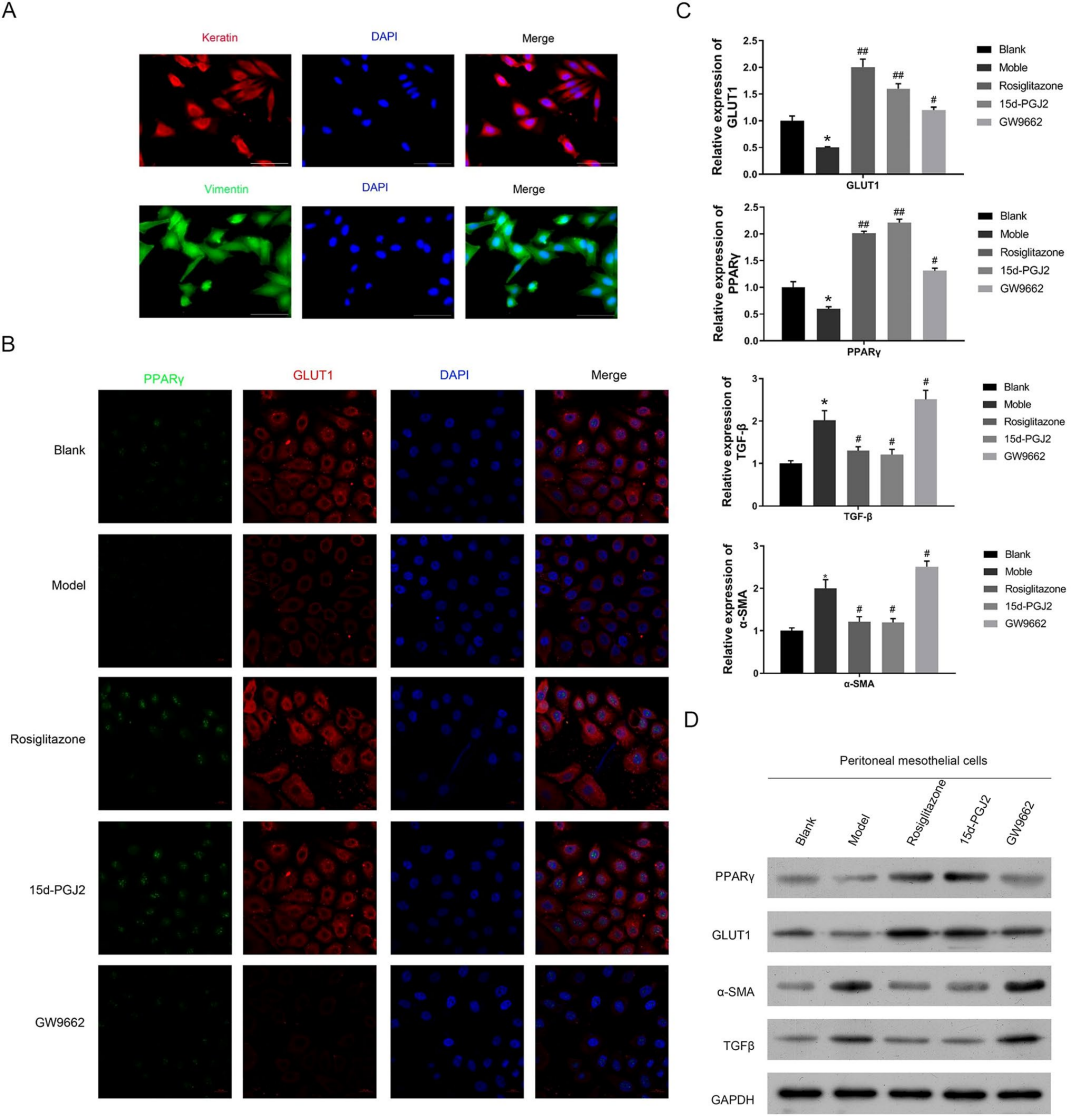
Modulation of GLUT1 expression and fibrosis in rat peritoneal mesothelial cells by PPARγ. **A** Expression of two mesothelial markers keratin and vimentin proteins in peritoneal mesothelial cells isolated from rat PF models treated with or without PPARγ agonist/antagonist. The expressions of keratin and vimentin proteins in peritoneal mesothelial cells were evaluated by immunofluorescence method. **B** Subcellular distribution and expressional alterations of PPARγ and GLUT1 proteins in rat peritoneal mesothelial cells after PPARγ agonist/antagonist treatments. PPARγ and GLUT1 protein expression and localization were detected through laser confocal microscopy. **C** Relative mRNA levels of PPARγ, GLUT1, TGF-β, and a-SMA gene in peritoneal mesothelial cells isolated from rats under PPARγ agonist/antagonist treatments. The levels of mRNAs were relatively measured by the quantitative RT-PCR method. **D** PPARγ, GLUT1, TGF-β, and a-SMA protein levels in peritoneal mesothelial cells from rats treated with PPARγ agonist/antagonist. Western blotting assay was performed to analyze protein abundance, with GAPDH detected as the internal standard. *PPAR*γ peroxisome proliferator-activated receptor gamma, *GLUT1* glucose transporter type 1, *TGF-β* tumor growth factor beta, *a-SMA* a-smooth muscle actin, *GAPDH* glyceraldehyde-3-phosphate dehydrogenase; *P < 0.05 (vs the blank group); ^#^*P* < 0.05 and ^##^*P* < 0.01 (vs the model group)

For further validation of the roles of PPARγ in regulating GLUT1 expression and peritoneal fibrosis development, the primary culture of peritoneal mesothelial cells was prepared from the above-mentioned rat PF models treated with or without PPARγ agonist/antagonist. At first, the identities of isolated rat peritoneal mesothelial cells were confirmed by the significant expression of two mesothelial markers, keratin and vimentin proteins, which were detected by immunofluorescence (Fig. 3A). Subsequently, we showed using laser confocal fluorescence method that PPARγ proteins were mainly distributed in the nucleus of rat peritoneal mesothelial cells, while the GLUT1 proteins were mainly localized in the cytosol components of the rat peritoneal mesothelial cells (Fig. 3B). Moreover, both the expressions of PPARγ and GLUT1 proteins in rat peritoneal mesothelial cells from the model group were markedly lowered compared with the blank group, but significantly increased by rosiglitazone or 15d-PGJ2 and greatly reduced by GW9662 treatment (Fig. 3B). Moreover, we also confirmed consistent changes of PPARγ and GLUT1 expression in rat peritoneal mesothelial cells following rosiglitazone/15d-PGJ2/GW9662 treatment by both the quantitative RT-PCR and western blotting methods (Fig. 3C and D). Furthermore, the expressions of two fibrogenesis marker genes a-SMA (a-smooth muscle actin) and TGF-β (tumor growth factor beta) in the rat peritoneal mesothelial cells of the model group, which were greatly higher than those from the blank group, were significantly repressed by rosiglitazone/15d-PGJ2 treatment and effectively elevated by GW9662 treatment (Fig. 3C and D).

### PPARγ suppresses the fibrosis and proliferation of rat peritoneal mesothelial cells.

**Fig. 4:**
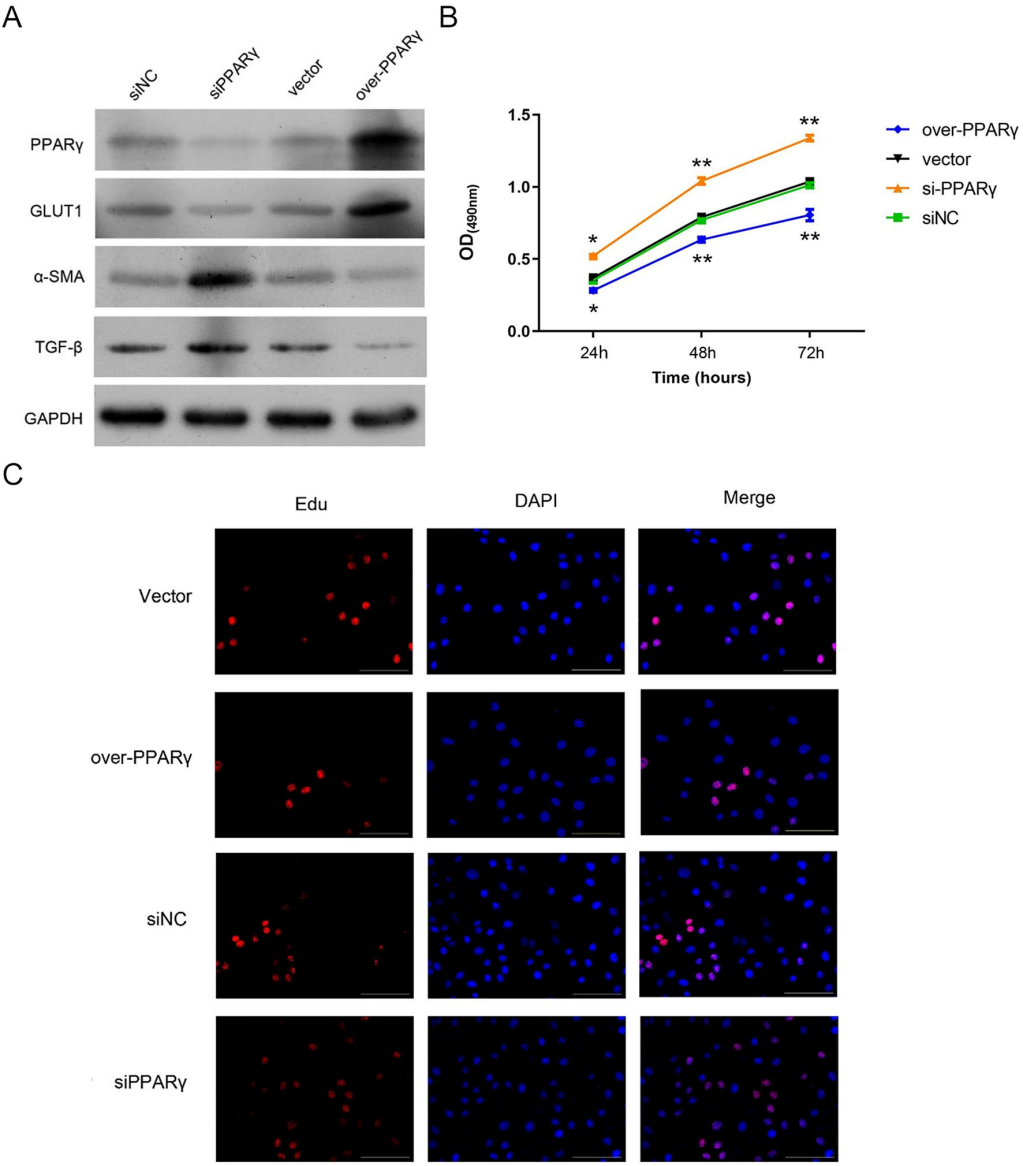
Regulation of rat peritoneal mesothelial cell fibrogenesis and proliferation by PPARγ siRNAs and overexpression. **A** Alterations of PPARγ, GLUT1, TGF-β, and a-SMA protein contents in rat peritoneal mesothelial cells with silenced or overexpressed PPARγ gene. PPARγ overexpression was induced by transfection with the recombinant H125-PPARγ lentivirus vector, in which PPARγ silencing was realized by transfecting the si PPARγ. Protein levels in peritoneal mesothelial cells were assessed via the western blotting with GAPDH detected as the internal standard. **B**, **C** Influences of PPARΓγ gene silencing and overexpression on the proliferation of rat peritoneal mesothelial cells. The proliferation rates of rat peritoneal mesothelial cells were analyzed by both the MTT (**B**) and Edu staining (**C**) methods. *siNC* siRNA negative control, *siPPARγ* PPARγ siRNAs, *PPARγ* peroxisome proliferator-activated receptor gamma, *GLUT1* glucose transporter type 1, *TGF-β* tumor growth factor beta, *a-SMA* a-smooth muscle actin; OD (450): absorbance at 450 nm, *GAPDH* glyceraldehyde-3-phosphate dehydrogenase; *P < 0.05; **P < 0.01

To verify the effects of PPARγ on GLUT1 expression, fibrosis, and proliferation of peritoneal mesothelial cells, we finally established the rat peritoneal mesothelial cells with PPARγ overexpression using the recombinant lentivirus transfection or PPARγ knockdown mediated by siPPARγ transfection. We observed that PPARγ gene knockdown with siRNAs caused great decreases of PPARγ and GLUT1 expression, as well as significant increases of TGF-β and a-SMA gene expression in rat peritoneal mesothelial cells. Contrarily, the expressions of PPARγ and GLUT1 were significantly promoted, and the expressions of TGF-β and a-SMA were remarkably down-regulated by PPARγ overexpression in rat peritoneal mesothelial cells (Fig. 4A). Our following MTT assay showed that the proliferation of rat peritoneal mesothelial cells was significantly promoted by PPARγ siRNAs and remarkably repressed by PPARγ overexpression (Fig. 4B). In consistence, we also observed from Edu staining the PPARγ siRNAs promoted rat peritoneal mesothelial cell proliferation, while PPARγ overexpression resulted in significant suppression of rat peritoneal mesothelial cell proliferation (Fig. 4C). Together, these results further validated that PPARγ exerts inhibitory effects on the fibrogenesis and proliferation of rat peritoneal mesothelial cells.

### Overexpression of PPARγ inhibits peritoneal fibrosis progression in rat model

**Fig. 5:**
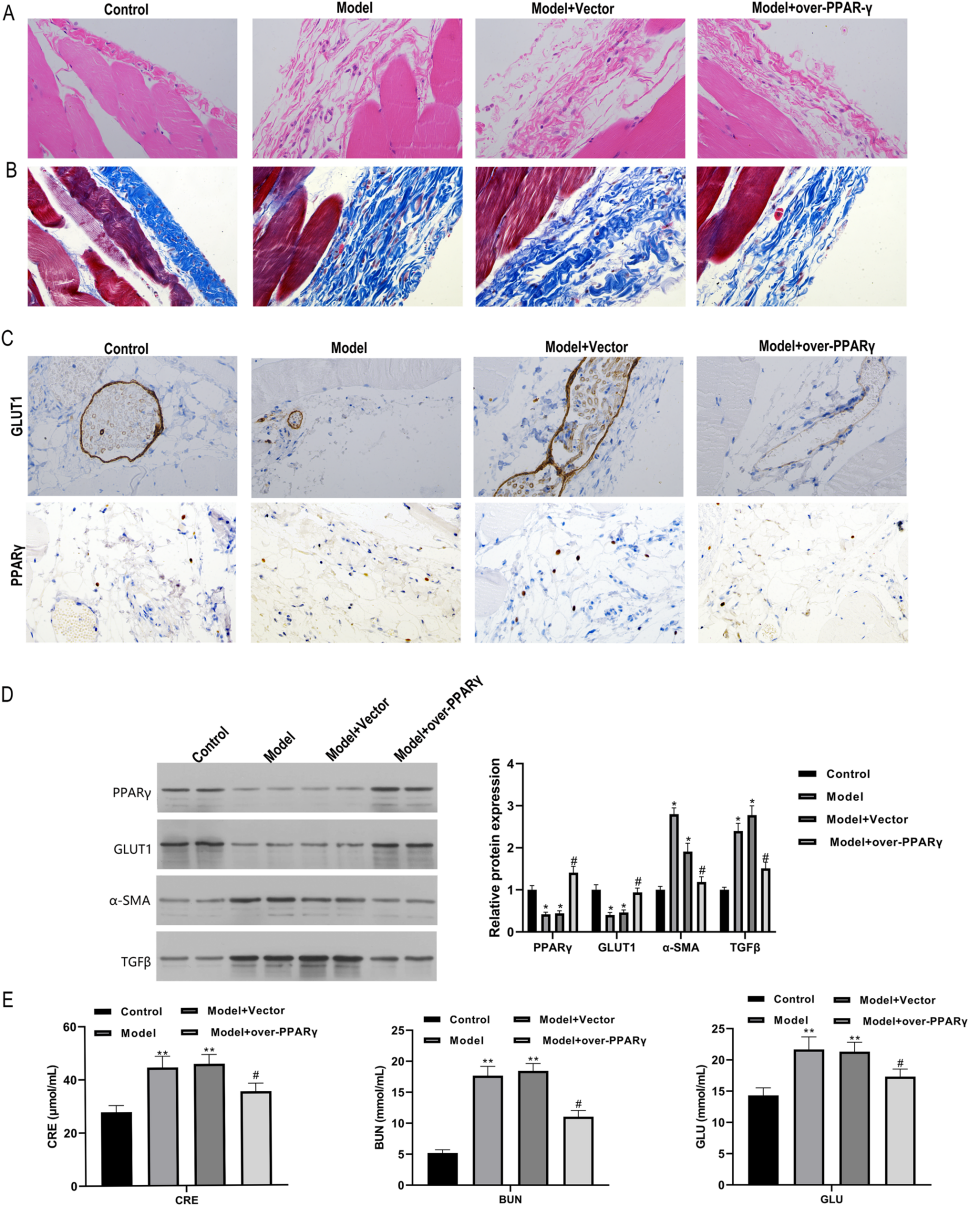
Overexpression of PPARγ inhibits peritoneal fibrosis progression in rat model. **A** Histological evaluation of the influences of overexpression of PPARγ in the rat of different groups. Rat peritoneal tissues were subjected to hematoxylin–eosin (H&E) staining and observed under microscopy. **B** The changes of collagen fiber contents in the peritoneal membranes of rat PF model treated with overexpression of PPARγ. Collagen fiber levels in rat peritoneal membranes were measured by Masson staining. **C** In situ PPARγ and GLUT1 protein levels in the peritoneal membranes of rat PF model affected by treatment with overexpression of PPARγ. Immunohistochemistry method was used to detect the in situ expression of PPARγ and GLUT1 in rat peritoneal membrane. **D** PPARγ, GLUT1, TGF-β, and a-SMA protein levels in the peritoneal membranes of rat PF model treated with overexpression of PPARγ. Western blotting assay was performed to analyze protein abundance, with GAPDH detected as the internal standard. **E** The alteration of creatinine, urea nitrogen, and blood glucose contents in the rat PF model treated with overexpression of PPARγ. The rat creatinine, urea nitrogen, and blood glucose levels of each group as indicated were determined through the ELISA method. *P < 0.05 and **P < 0.01 (vs the control group); ^#^*P* < 0.05 and ^#^*P* < 0.05 (vs the model + vector group)

To further explore the role of PPARγ in regulating the development of peritoneal fibrosis (PF), we constructed PPARγ overexpression vector and injected it into PF rats. We observed that the thickness of the rat submesothelial layer in the model group was greatly increased compared with the control group, which was then remarkably decreased by PPARγ overexpression treatment (Fig. 5A). In addition, Masson staining demonstrated that the collagen fiber content in the peritoneal membrane tissues of the rat model group was also greatly elevated in comparison to the control group, which was significantly reduced by PPARγ overexpression treatment (Fig. 5B). Furthermore, the promotion of GLUT1 and PPARγ protein level by PPARγ overexpression in peritoneal membranes of rat PF model were all verified by the immunohistochemistry (IHC) assay (Fig. 5C). In consistence, overexpression of PPARγ significantly inhibited a-SMA and TGF-β as well as promoted the expression of PPARγ and GLUT1 in the peritoneum of model rats (Fig. 5D). As expected, the creatinine, blood urea nitrogen, and blood glucose contents in the model rats were significantly decreased by Overexpression of PPARγ treatment compared with the model group (Fig. 5E). However, the PPARγ overexpression effectively decreased CRE, BUN, and blood glucose levels in the PF model rats (Fig. 5E). These results showed that PPARγ overexpression alleviated PF progression in the rat model.

## Discussion

The development of peritoneal fibrosis (PF) caused by long-term application of peritoneal dialysis solution has long been a severe medical problem impeding clinical management of patients with multiple renal diseases [3, 5]. The aberrant changes of peritoneal membrane morphology and physiology during PF pathogenic process, especially the peritoneal mesothelial cells, are known to mediated by complex cellular and molecular pathways, such as GLUT1-mediated glucose transport and energic metabolism [6, 10]. However, the up-stream regulatory components responsible for affecting GLUT1 expression in peritoneal mesothelial cells and PD-induced PF progression remain poorly understood. In the present study, we reported that PPARγ could substantially modulate peritoneal fibrosis pathogenesis in rat model. Importantly, we showed here that for the first time that GLUT1 in rat peritoneal membrane tissues and peritoneal mesothelial cells were positively regulated by PPARγ. Contrarily, the expression of a-SMA and TGF-b showed significant negative correlation with PPARγ and GLUT1 in rat peritoneal mesothelial cells. Moreover, PPARγ silencing or overexpression could effectively suppress the proliferation of rat peritoneal mesothelial cells and fibrosis. These observations disclosed new molecular mechanisms driving the peritoneal mesothelial cell functional alterations associated with PF pathogenesis.

Due to the pleiotropic roles of PPARγ protein in various biological and physiological processes, the screening and application of agonists or antagonist targeting PPARγ have been a research hotspot for disease treatment in recent decades [19–21]. For instance, the development of glomerulosclerosis and renal fibrosis associated with TGF-β activation could be significantly repressed by the treatment with one PPARγ agonist pioglitazone [15]. In terms of PD-induced peritoneal fibrosis, previous reports using both animal and cellular models demonstrated the effective suppression of PD/high glucose-induced PF development by treatments with PPARγ agonists such as Telmisartan and pioglitazone [16, 17]. In this report, we showed that PPARγ expression was greatly reduced in the rat PF model, and PPARγ agonists rosiglitazone and 15d-PGJ2 repressed PF progression, while PPARγ antagonist GW9662 promoted PF development in rat model. α-SMA (α-smooth muscle actin), as one key cytoskeleton protein component associated with fibrogenesis process [22], and TGF-β, which acts as one major inflammatory factor and the master regulator of fibrogenesis in various contexts [23, 24], have been widely applied as two main biomarkers of fibrogenic pathogenesis. We also revealed that α-SMA and TGF-β expression in rat peritoneal mesothelial cells were suppressed by PPARγ agonists rosiglitazone and 15d-PGJ2 but elevated by PPARγ antagonist GW9662, which further validated the fibrosis-regulating roles of PPARγ. Furthermore, the potentials of rosiglitazone and 15d-PGJ2, as well as other PPARγ agonists, in treating PD-associated peritoneal fibrosis deserve further explorations through large-scale clinical trials.

GLUT1 is a transmembrane glucose transporter implicated in the development of multiple fibrosis-related human disorders such as renal fibrosis, liver fibrosis, and peritoneal fibrosis [8, 9]. More importantly, the expression of GLUT1 gene could be promoted by PPARγ during the modulation of renal tubule homeostasis, thus being essentially implicated in the progression of renal tubulointerstitial fibrosis [18]. However, little is known about the interaction between PPARγ and GLUT1 expression in peritoneal mesothelial cells and PD-induced PF pathogenesis. Like the PPARγ expression, we found here that GLUT1 expression was also repressed in the rat PF model. Also, we proved that the expression of GLUT1 gene in both the rat peritoneal membrane tissues and isolated rat peritoneal mesothelial cells were greatly elevated by PPARγ agonists rosiglitazone and 15d-PGJ2 and repressed by PPARγ antagonist GW9662. Also, PPARγ siRNA down-regulated GLUT1 gene expression, while PPARγ overexpression greatly increased GLUT1 gene expression in rat peritoneal mesothelial cells. These results clearly demonstrated the positive modulation of GLUT1 expression in rat peritoneal mesothelial cells associated with high glucose-induced PF development. Through immunofluorescence combined with laser confocal microscopy, we showed that PPARγ and GLUT1 proteins were mainly localized in the nucleus and cytosols of rat peritoneal mesothelial cells, respectively. It is plausible that PPARγ, as a transcription factor, might directly regulate the transcription of GLUT1 gene during PF pathogenesis, which deserves further elucidations.

As introduced above, the fibrogenesis and PF development was closely associated with the aberrant functional changes in the peritoneal mesothelial cells, such as their proliferation, apoptosis, and even epithelial-to-mesenchymal transition [5, 25, 26]. It has been already known that the proliferation of peritoneal mesothelial cells could be affected by the Y-box-binding protein 1 (YBX1) and Twist1 proteins during the development of peritoneal fibrosis caused by high glucose [27]. More importantly, repressing the proliferation of peritoneal mesothelial cells has been shown as a promising way of inhibiting peritoneal fibrosis development induced by high glucose and dialysis. For instance, the anti-proliferative and anti-fibrotic chemical dipyridamole significantly repressed PD-induced PF development through inhibiting peritoneal mesothelial cell proliferation in both in vitro and in vivo assays [28, 29]. In the present study, we demonstrated that PPARγ overexpression effectively decreased the proliferation rates of rat peritoneal mesothelial cells, while PPARγ siRNAs caused great increase of rat peritoneal mesothelial cell proliferation. These observations further highlighted the significance of peritoneal mesothelial cell proliferation in the pathogenesis of PD-induced peritoneal fibrosis. More importantly, these results suggested that the regulation of peritoneal mesothelial cell proliferation by targeting PPARγ expression might serve as a potential target for treating PF patients following peritoneal dialysis.

Taking together, we unveiled in this study that PPARγ significantly repressed the progression of peritoneal dialysis-induced peritoneal fibrosis in rat PF model established by high glucose, which was mediated by the elevation of GLUT1 protein expression to suppress peritoneal mesothelial cell proliferation. These investigations disclosed new insights into the pathogenic mechanisms underlying high glucose-induced peritoneal fibrosis, which might also serve as a basis for developing new prevention and treatments for peritoneal fibrosis in patients undergoing peritoneal dialysis.

## Data Availability

The data obtained in this research are available from the corresponding author on reasonable request.
